# Function or Cosmesis—What Is the Predominant Concern in Patients With Nasal Trauma Presenting for Rhinoplasty?

**Published:** 2009-03-03

**Authors:** Carl M. Philpott, Allan Clark, David C. McKiernan

**Affiliations:** ^a^ Departments of Otorhinolaryngology, Cambridge University Hospitals, (Addenbrooke's and West Suffolk Hospitals) NHS Trusts, Cambridge, United Kingdom; ^b^ School of Medicine, Health Policy and Practice, University of East Anglia, Norwich, NR4 7TJ United Kingdom

## Abstract

**Objectives:** To assess whether or not patients receiving rhinoplasties following nasal trauma sought intervention for a functional or cosmetic reason and look at underlying psychosocial influences. **Methods:** A cross-sectional questionnaire study was performed in the setting of otorhinolaryngology outpatient clinics in the Cambridge University Hospitals. New patients referred to the clinic with nasal deformities secondary to recent trauma were included. To provide a control group, new patients attending for nonrhinological reasons were also asked to complete 2 questionnaires (a study specific one and the SF36). The age range of patients was 21 to 66 years in the control group and 17 to 67 years in the rhinoplasty group. **Results:** Patients attending for rhinoplasty were more likely to be male (79% vs 37%, ***P*** = .008) and have had previous nasal trauma (relative risk = 2.14, ***P*** = .0086) They neither had significantly higher scores for the SF36 or higher alcohol consumption nor were more likely to participate in contact sports than the control group nor did they differ significantly in terms of social class. **Conclusion:** This study did not find evidence that posttrauma rhinoplasty patients are anymore introspective and depressed than the normal control population and that function was the predominant concern over cosmesis.

Patients requiring rhinoplasty are commonly seen in ENT clinics for assessment. The psychological profile of patients with nontraumatic cosmetic deformities seeking rhinoplasty has been studied before,^1^,^2^ but the specific traits in patients suffering nasal trauma seeking rhinoplasty has not. The benefits of operating on patients for the reason of cosmesis over that of function has been demonstrated to be greater in a study by McKiernan et al.^3^ Rhinoplasty patients do not appear to have a different perception of the ideal nose,^4^ but may have inherently different psychosocial factors that predispose them to trauma in the first place or lead them to seek intervention more readily than others. The psychological outcomes postoperatively following rhinoplasty have been shown generally to be positive^5^–^10^ except when factors such as having unrealistic expectations of the procedure, previous unsatisfactory cosmetic surgery, minimal deformity, and motivation based on relationship issues exist.^11^–^13^ Not surprisingly, a history of depression, anxiety, personality disorder, or body dysmorphic disorder are predictors of dissatisfaction with the postoperative result, but factors that may not be widely recognized include younger age or male sex;^11^,^14^–^16^ however, it is women who are more prone to suffer psychological impairment when a cosmetic deformity of the nose exists.^2^

The specific psychological profile of **posttrauma** patients seeking rhinoplasty has not been studied to date in the United Kingdom. The focus of other studies, excepting one discussed below,^17^ has been cosmesis. Patients with developmental deformities have been shown to be less likely to choose rhinoplasty independently after the age of 21,^18^ and the effect of external influences in the trauma patients is unknown. Nontraumatic cases have also been shown to have higher psychiatric morbidity.^19^,^20^ A Swedish study conducted almost 20 years ago suggested a higher rate of alcohol consumption and susceptibility to injury in the trauma cases.^17^ With increasing trends of binge drinking, especially in UK youth culture, this is clearly an area that needs revisiting. This study aims to assess both psychological and social traits in patients referred to National Health Service otolaryngology clinics with posttraumatic nasal deformity seeking rhinoplasty.

## SUBJECTS AND METHODS

**Figure 1:**
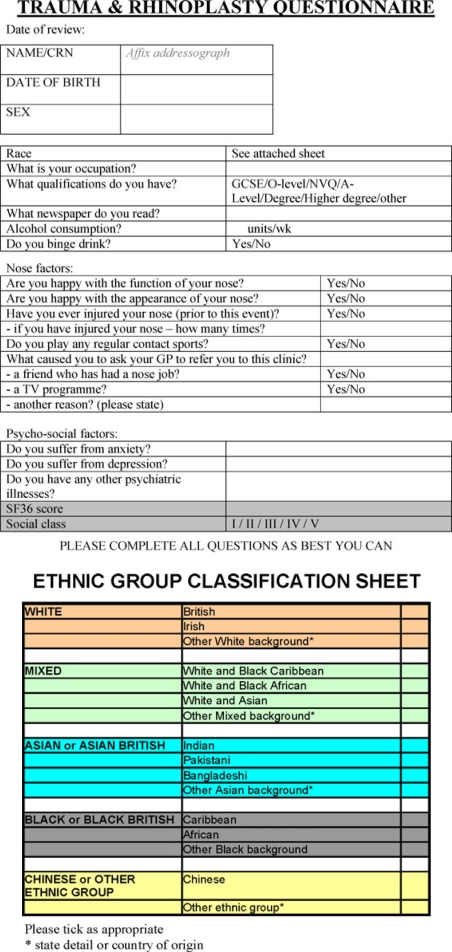
Patient questionnaire.

This was a questionnaire-based study with patients being selected to complete questionnaires at the time of their outpatient appointment in the ENT Clinics at Bury St Edmunds, Newmarket, and Cambridge. New referrals for nasal deformity secondary to trauma were invited to complete the questionnaire (Fig 1) that incorporated the Short Form 36 Quality of Life Questionnaire.^21^ To provide a control group, new nonrhinological referrals were also invited to complete the questionnaire in the same setting. Patients who were unable to comprehend English or had nasal deformity due to a nontraumatic etiology were excluded. Patients were excluded from the control group if they had any concomitant rhinological problems. Thirty-eight patients' questionnaires were collected—19 in each group. These were then collated and the data analyzed by using Stata software (Stata SE for Windows, Version 9.1, College Station, Tex).

## RESULTS

**Figure 2:**
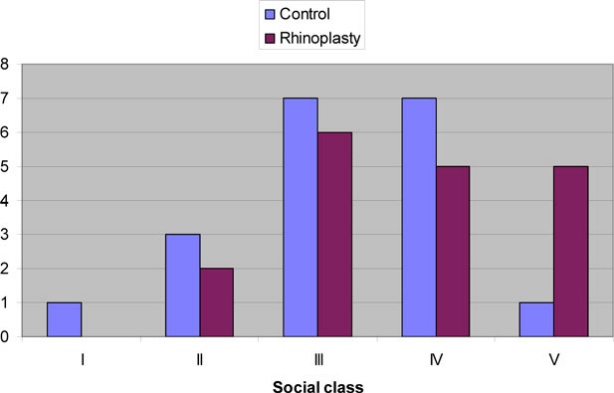
Correlation with social class.

**Figure 3:**
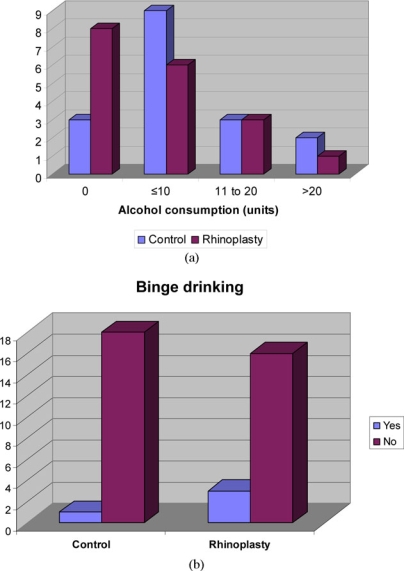
(a) and (b) Correlation with alcohol consumption and binge drinking.

**Figure 4:**
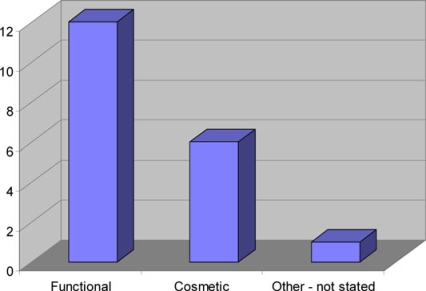
Reason for referral to the ENT clinic.

The age range of patients was 21 to 66 years in the control group and 17 to 67 years in the rhinoplasty group (means of 42 and 33, respectively). Patients referred to the outpatient clinic for consideration of rhinoplasty showed the expected positive correlation with unhappiness regarding their nasal function and cosmesis (*P* ≤ .001 and .0033, respectively; Table 1). They were also more likely to have injured their nose on a previous occasion (*P* = .0086). There was a greater proportion of males with nasal trauma in the rhinoplasty group than in the control group (*P* = .008). There was no evidence of a difference in social class between the 2 groups (*P* = .373) (Fig 2). However, the other variables considered did not demonstrate any significant differences between the 2 groups. Alcohol consumption did not have a positive correlation with nasal trauma, and binge drinking was only admitted by 4 patients although 3 of these were in the rhinoplasty group (Figs 3a and 3b). In fact there were more “teetotallers” in the rhinoplasty group than the control group. There was also no significant difference in quality of life as assessed by the SF36 questionnaires. The predominant reason these patients stated for referral to the ENT clinic was to restore nasal function (60%) (Fig 4).

## DISCUSSION

A functional defect in these patients' noses resulting from trauma was the predominant feature for being referred by their general practitioner to the clinic (60% of rhinoplasty patients). This observation is interesting because it suggests that patients who have suffered nasal trauma are far more concerned about rectifying the functional problems of their nose over the cosmetic ones. High alcohol intake, especially in male patients, poorly established relationships, and an asthenic temperament have been shown in previous studies to be psychosocial aspects associated with these patients and with a poor outcome from rhinoplasty.^17^,^22^ When Kurtzberg et al^22^ studied a number of prison inmates with facial disfigurements in 1967, compared with their fellow inmates who had not had surgery, those who underwent facial plastic surgery had improved psychosocial adjustment and significant lower recidivism rates. It might be anticipated that alcohol abuse and depression are more common in the lower social classes, but the findings of these previous studies were not echoed by any associations with alcohol in our study and may well be a reflection of the skewed picture represented by studying prison inmates in Kurtzberg's study, although patients may be reluctant or embarrassed to admit to excessive alcohol consumption to a doctor. It was however evident in our study that more of the rhinoplasty patients were from social classes IV and V, although this was not statistically significant. As this study excluded private patients, it may be that more patients of higher social class seek treatment for posttraumatic nasal deformity privately because Trusts are increasingly perceived to regard this surgery as “low priority.”

In this study, patients had newly acquired posttraumatic nasal deformities and a psychological impact was not identified in them, judging by their responses to the SF36s. Individual psychological ideas are influenced by ethnic and cultural stereotypes, which may be partly reflected by their social class. In terms of ethnicity, our patient groups were predominantly of British white ethnic origin, with only 10% being of other white or mixed ethnic origin (all in the rhinoplasty group). This reflects the predominantly white racial group of the local population that our hospitals serve in Western Suffolk and Eastern Cambridgeshire in the United Kingdom.

In the 1990s, a study performed to look at the perception of attractiveness in facial features found that the faces examined deemed by the subjects as most attractive were not average in terms of facial structure.^23^ This helps convey the concept that self-perception of facial features can be a complex psychological issue. Clearly, there are proven psychological benefits to undertaking cosmetic nasal surgery,^7^,^24^ provided that patients with clear evidence of body dysmorphic order are excluded^14^ and this is to be found in 5% of patients seeking rhinoplasty.^25^ The assessment of expectations, motivations, and concerns often reveals useful information and the surgeon should be wary of patients who have had previous unsuccessful operations or consultations. Grey areas can appear where a patient presents to the clinic with a posttraumatic nasal deformity such as deviated nasal bones, but wishes to have a preexisting nasal deformity corrected at the same time. Psychological assessment or support should be utilized when there are any doubts as to the appropriateness of surgical intervention.

## CONCLUSIONS

The significant relationships demonstrated in our study were for male sex, and previous nasal injury, and nasal function was of greater concern than cosmesis to the rhinoplasty patients. Other factors may not have been demonstrated in our study owing to the relatively small number of patients included. Further studies with larger numbers may well demonstrate significant correlations, especially in respect to alcohol consumption. This study however presents a relatively unique look at patients presenting for rhinoplasty after nasal trauma alone rather than those with developmental deformities.

## Figures and Tables

**Table 1:** Analysis of results

	Nasal group		Control group				
Variable Total	*N* 19	%	*N* 19	%	*P*	95% Confidence interval	*R*^2^
Gender							
Male	15	78.9	7	36.8	.0086	1.14—4.03	2.14
Female	4		12				
Education							
A-level or degree	7	38.9	2	15.4	.1548	0.62–10.25	2.52
GCSE or less	11		11				
Binge drinking							
Yes	3	15.8	1	5.3	.6039	0.34–26.33	3
No	16		18				
Social class					.373		
I	0		1				
II	2		3				
III	6		7				
IV	5		7				
V	5		1				
SF-36							
Score	74.22		75.42		.8412	–10.86–13.26	
mean (SD)	(17.84)		(18.5)				
Injured nose							
Yes	15	78.9	7	36.8	.0086	1.14–4.03	2.14
No	4		12				
Cosmesis							
Yes	6	31.6	15	78.9	.0033	0.20–0.81	0.4
No	13		4				
Function							
Yes	3	15.8	16	84.2	<.0001	0.07–0.54	0.1875
No	16		3				

GCSE indicates General Certificate of Secondary Education.
